# Effects of switching from clopidogrel to prasugrel at the chronic phase after coronary stenting on antiplatelet action and vascular endothelial function: Switch-Pras study

**DOI:** 10.1007/s00380-020-01714-w

**Published:** 2020-10-28

**Authors:** Taiki Masuyama, Masashi Sakuma, Ryutaro Waku, Suguru Hirose, Keijiro Kitahara, Jin Naganuma, Hiroko Yazawa, Shigeru Toyoda, Shichiro Abe, Toshiaki Nakajima, Teruo Inoue

**Affiliations:** Department of Cardiovascular Medicine, School of Medicine, Dokkyo Medical University, 880 Kitakobayashi, Mibu, Tochigi 321-0293 Japan

**Keywords:** Prasugrel, Clopidogrel, Platelet reactivity, Endothelial progenitor cell, Coronary artery disease

## Abstract

Compared to clopidogrel, prasugrel has a lower incidence of ischemic events following percutaneous coronary intervention (PCI) because of an early reduction during the acute phase in P2Y12 reaction units (PRU). The objective of this study was to compare the antiplatelet effect and vascular endothelial function of both drugs during the chronic phase after PCI. Patients who had undergone PCI and were confirmed to have no restenosis by follow-up coronary angiography under dual anti-platelet therapy with clopidogrel (75 mg/day) and aspirin (100 mg/day) were randomized to either continue clopidogrel or switch to prasugrel (3.75 mg/day). At baseline, prior to randomization we determined the CYP2C19 genotype. At the baseline and 24 weeks after randomization, the P2Y12 reactivity unit (PRU) was measured using the VerifyNow™ P2Y12 assay. Endothelial function was evaluated by flow-mediated vasodilation (FMD) and reactive hyperemia peripheral arterial tonometry (RH-PAT), while and circulating CD34+/CD133+/CD45^low^ progenitor cells were measured by flow cytometric analysis. Serum high-sensitivity C-reactive protein (hsCRP) level was also measured. The PRU was reduced significantly in the prasugrel group (*P* = 0.0008), especially in patients who were intermediate or poor metabolizers based on the CYP2C19 genotype (*P* < 0.0001). This reduction was not observed in the clopidogrel group. The number of CD34+/CD133+/CD45^low^ cells increased in the clopidogrel group (*P* = 0.008), but not in the prasugrel group. The hsCRP, FMD and reactive hyperemia index measured by RH-PAT did not change in either group. Prasugrel is potentially better than clopidogrel for preventing thrombotic events, although clopidogrel may have an advantage over prasugrel in terms of preventing atherosclerotic events. Proper use of thienopyridine drugs based on the CYP2C19 genotype has promising clinical potential.

## Introduction

Thienopyridines, a class of selective irreversible inhibitors of the adenosine diphosphate receptor, P2Y12, have been used widely for their antiplatelet action [1]. Dual anti-platelet therapy using thienopyridines along with aspirin is the gold standard treatment in patients with the acute coronary syndrome (ACS) and/or patients undergoing percutaneous coronary intervention (PCI) to prevent coronary events such as recurrence of ACS and/or stent thrombosis. Of the different types of thienopyridines, clopidogrel was used most frequently to prevent coronary events until several years ago [2]. However, recently an alternative thienopyridine, prasugrel, has been developed which provides stronger platelet inhibition than clopidogrel [3]. Two recent Japanese trials, PRASFIT-ACS (PRASugrel compared with clopidogrel For Japanese patienTs with ACS undergoing PCI) [4] and PRASFIT-Elective (PRASugrel For Japanese PatIenTs with Coronary Artery Diseases Undergoing Elective PCI) [5], demonstrated that prasugrel tended to reduce mid-term (24 weeks) ischemic events in patients with ACS as well as stable coronary artery disease who underwent PCI to a greater extent than that observed with clopidogrel. Moreover, analysis of 1-year follow-up data from a post-marketing observational study, PRASFIT-Practice II (Prasugrel for Japanese Patients with Ischemic Heart Disease in Long-Term Clinical Practice (PRASFIT-Practice II) [6], showed long-term effectiveness and safety of prasugrel at dosages approved in Japan for the treatment of coronary artery disease patients undergoing PCI. It is likely that prasugrel causes a greater reduction in platelet reactivity than clopidogrel, the action of which may be affected by genetic variants of the cytochrome P450 enzyme, CYP2C19. However, there is little information as to whether prasugrel has greater benefits than clopidogrel on longer-term outcomes after PCI.

In the drug-eluting stent (DES) era the majority of mid-term ischemic events are stent thrombosis. Therefore, to improve long-term ischemic outcomes it is necessary to prevent not only late thrombosis but also late restenosis (late catch-up) [7], neoatherosclerosis [8] and impaired vascular healing [9] of the target lesion. It is also essential to prevent the atherosclerotic process in the entire coronary artery system beyond the target lesions [10]. It has been suggested that thienopyridines have direct pleiotropic anti-atherosclerotic effects independent of their anti-platelet action, including improvement of vascular endothelial function [11], an anti-inflammatory action [12] and reduction of oxidative stress [13].

The present study in patients with coronary artery disease, the Switch-Pras study, was designed to investigate whether switching from clopidogrel to prasugrel during the late phase after PCI had beneficial effects in terms of subsequent platelet reactivity, mobilization of endothelial progenitor cells (EPCs) and vascular endothelial function.

## Methods

### Study design

**Fig. 1:**
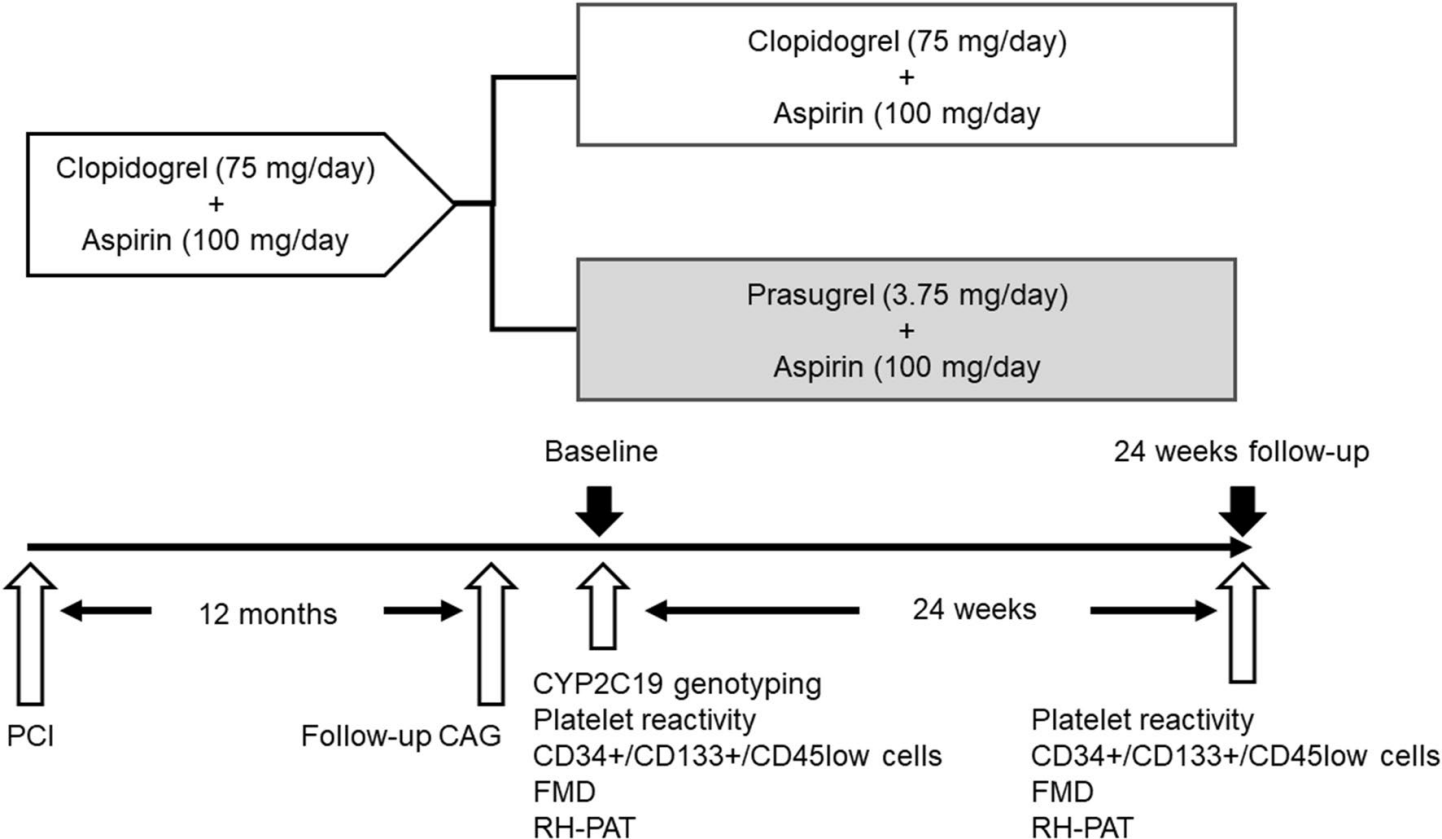
Study protocol

The study was a prospective randomized controlled design. The study enrolled 100 patients with coronary artery disease who had received dual anti-platelet therapy with proton pump inhibitor (20 mg/day esomeprazole) after a PCI (100 mg/day aspirin and 75 mg/day clopidogrel) and were confirmed to have no stenotic lesions in target vessels in follow-up coronary angiography carried out 12 months later. After follow-up coronary angiography, the patients were assigned randomly using a computer-based random number table to continue receiving clopidogrel (clopidogrel group; *n* = 50) or switch to 3.75 mg/day prasugrel (prasugrel group; *n* = 49). Both treatments were combined with 100 mg/day aspirin and 20 mg/day esomeprazole. The patients were then followed-up for 24 weeks (Fig. 1). At baseline and before randomization we collected data of demographic and clinical characteristics, carried out a genetic analysis of the CYP2C19 polymorphism and performed clinical tests to assess platelet reactivity, to estimate the number of circulating CD34+/CD133+/CD45^low^ cells as an EPC linage, to measure the level of high sensitivity C-reactive protein (hsCRP) as an inflammatory biomarker, and to assess vascular endothelial function. The study was approved by the Dokkyo Medical University Hospital review board and was conducted in full compliance with the Declaration of Helsinki and Ethical Guidelines for Medical and Health Research Involving Human Subjects established by the Ministry of Health, Labor, and Welfare. The details of the study were registered with the University Hospital Medical Information Network clinical trial registry (No. UMIN 000027321).

### Genetic analysis

The genetic analysis was performed using the QIAamp Blood Kit (Qiagen, Hilden, Germany) to isolate genomic DNA. Genotyping of CYP2C19 single-nucleotide polymorphisms (SNP; 681G > A and 636G > A) was performed using the Invader DNA assay method (Third Wave Technologies, Madison, WI, USA). The SNP genotype was translated into a star-allele genotype and the patients classified into 3 phenotypes: extensive metabolizer (EM; *1/*1), intermediate metabolizer (IM; *1/*2, *1/*3), and poor metabolizer (PM; *2/*2, *2/*3, *3/*3) [14].

### Platelet reactivity testing

Platelet reactivity was assessed using the VerifyNow^®^ assay kit (Accumetrics, San Diego, CA, USA) according to the manufacturer’s instructions. Blood samples were collected in buffered sodium citrate tubes 8–24 h after intake of the antiplatelet agents, primarily in the morning after an overnight fast and a rest period of at least 20–30 min. Careful antecubital venipuncture was performed by an experienced nurse or physician using a 21-gauge syringe. The VerifyNow^®^ assay measures adenosine diphosphate-induced platelet function as an increase in light transmittance expressed as P2Y12 reaction units (PRU) [15].

### *Measurement of circulating CD34+/CD133+/CD45*^*low*^* cells*

Circulating CD34+/CD133+/CD45^low^ cells, including EPCs were measured by flow cytometry using minor modifications of a previously described method [16–18]. In brief, EDTA-treated peripheral blood was incubated with the test or control reagent. The reagent mixture consisted of nucleic acid dye (SY-III-8; Molecular Probe, Eugene, OR, USA), peridinine chlorophil protein (PerCP)-conjugated anti-CD45 (Becton Dickinson, San Jose, CA, USA), fluorescein isothiocyanate (FITC)-conjugated anti-CD34 (Becton Dickinson) and phycoerythrin (PE)-conjugated anti-CD133 (Miltennyi Biotec, Bergisch Gladbach, Germany). Isotype controls were used as the negative controls based on the species and immunoglobulin (Ig) G control antibodies (IgG1 isotype control; Becton Dickinson). Flow cytometric analysis was then performed using a FACS Calibur laser flow cytometer (Becton Dickinson) according to the manufacturer’s instructions. Each measurement consisted of 10^6^ events of all white blood cells, which exceeded the threshold set for SY-III-8 fluorescence (nucleated cells). The absolute number of CD34+/CD133+/CD45^low^ cells per mL was calculated based on the cells-to-the whole-blood cell count.

### Measurement of high sensitivity C-reactive protein

The blood was centrifuged at 2000*g* for 10 min at room temperature and the serum samples were frozen and stored at −80 °C until analysis. The high sensitivity C-reactive protein (hsCRP) level was measured by particle-enhanced technology on the Behring BN II nephelometer (Dade Behring, Newark, DE, USA), using monoclonal anti-CRP antibodies and a calibrator that was traceable to WHO Reference Material [19].

### Vascular endothelial function testing

Brachial artery flow-mediated dilation (FMD) and reactive hyperemia-peripheral arterial tonometry (RH-PAT) were used to assess vascular endothelial function. Both procedures were performed simultaneously in the morning, according to the method previously described by Tomiyama et al. [20]; fasting overnight and abstaining from alcohol, smoking, caffeine and antioxidant vitamins for at least 12 h before the measurements. The patients were asked to rest in the sitting position in a quiet, dark, air-conditioned room (22 to 25 °C) for 5 min. They were then requested to rest again for at least 15 min in the supine position in the same room before the FMD and RH-PAT procedures. The FMD measurements were performed using UNEXEF18G (UNEX, Co, Nagoya, Japan), an ultrasound instrument specialized for FMD measurement. The RH-PAT procedure was carried out using an EndoPAT-2000 (Itamar Medical Ltd., Caesarea, Israel) to calculate the reactive hyperemia index (RHI).

### Statistical analysis

Normality for distribution of the continuous variables was assessed using the Shapiro–Wilk test. Values were expressed as the mean values ± standard deviation (SD) for parametric data and median values and interquartile ranges for non-parametric data. Intergroup comparisons were performed using unpaired *t* tests for parametric data and Mann–Whitney *U* tests for non-parametric data. Intragroup comparisons were carried out using paired *t* tests for parametric data and the Wilcoxon signed-rank test for non-parametric data. Inter-group comparisons of categorical variables were performed using the χ^2^ test. *P* < 0.05 was considered statistically significant.

## Results

### Baseline characteristics

Of the 100 enrolled patients, one patient in the prasugrel group was withdrawn because of discontinuation of prasugrel, leaving 99 patients in the full analysis set (clopidogrel group *n* = 50, prasugrel group *n* = 49). Baseline data for demographic and clinical characteristics were comparable in the two groups, with the exception that the incidence of hypertension and use of angiotensin-converting enzyme inhibitors/angiotensin receptor blockers and calcium channel blockers were higher in the clopidogrel group. Drug-eluting stents were used in 30 patients (60%) in the clopidogrel group and in 36 patients (73%) in the clopidogrel group. The period from PCI to follow-up coronary angiography was 14 ± 10 and 17 ± 10 months in the clopidogrel and prasugrel groups, respectively. Baseline platelet reactivity, number of CD34+/CD133+/CD45^low^ cells, hsCRP level, and vascular endothelial function were also compatible between the two treatment groups. In addition, the genetic analysis showed that the incidence of the CYP2C19 phenotype was similar in the two groups (clopidogrel, EM *n* = 20, IM *n* = 20, PM *n* = 10; prasugrel, EM *n* = 20, IM *n* = 23, PM *n* = 7; *P* = 0.684) (Table 1).

**Table 1:** Baseline characteristics

	Clopidogrel group (*n* = 50)	Prasugrel group (*n* = 49)	*P* value
Age (years)	68 ± 11	67 ± 9	0.624
Male gender, *n* (%)	39 (78)	42 (86)	0.320
Body mass index (kg/m^2^)	25 ± 4	24 ± 3	0.180
Underlying disease, *n* (%)			0.588
Stable angina pectoris	17 (34)	16 (33)	
Old myocardial infarction	33 (66)	33 (67)	
Affected vessel, *n* (%)			0.362
Single vessel disease	34 (68)	29 (59)	
Multi-vessel disease	16 (32)	20 (41)	
Period from PCI to follow-up CAG; months	14 ± 10	17 ± 10	0.126
Drug-eluting stent, *n* (%)	30 (60)	36 (72)	0.121
Risk factor, *n* (%)			
Hypertension	41 (82)	30 (61)	0.021
Diabetes	24 (48)	19 (39)	0.354
Dyslipidemia	36 (72)	35 (71)	0.950
Smoking	35 (70)	31 (63)	0.907
Systolic blood pressure (mmHg)	127 ± 16	127 ± 14	0.821
Diastolic blood pressure (mmHg)	72 ± 12	74 ± 10	0.293
Fasting blood glucose (mg/dL)	110 ± 25	114 ± 26	0.346
Hemoglobin A1c (%)	6.3 ± 0.8	6.2 ± 0.6	0.532
LDL-cholesterol (mg/dL)	81 ± 19	86 ± 19	0.221
HDL-cholesterol (mg/dL)	51 ± 12	53 ± 13	0.413
Triglyceride (mg/dL)	137 ± 86	125 ± 57	0.454
Creatinine (mg/dL)	0.84 ± 0.28	0.82 ± 0.17	0.646
eGFR (mL/min/1.73 m^2^)	72 ± 20	72 ± 16	0.896
Uric acid (mg/dL)	5.4 ± 1.2	5.4 ± 1.1	0.725
BNP (pg/mL)	38 ± 28	35 ± 30	0.572
hsCRP (mg/dL)	0.063 (0.029–0.135)	0.040 (0.021–0.080)	0.810
Medications, *n* (%)			
Statins	48 (96)	49 (100)	0,157
ACE inhibitors/ARBs	47 (94)	37 (76)	0.010
Beta blockers	29 (58)	24 (49)	0.368
Calcium channel blockers	27 (54)	16 (33)	0.368
Insulin	1 (2)	4 (8)	0.032
CYP2C19 phenotype, *n* (%)			0.684
Extensive metabolizer	20 (40)	19 (39)	
Intermediate metabolizer	20 (40)	23 (47)	
Poor metabolizer	10 (10)	7 (14)	
P2Y12 reaction unit	198 ± 65	192 ± 56	0.610
CD34+/CD133+ /CD45^low^ cell (cell/1 × 10^6^ WBC)	64 (48–98)	71 (46–96)	0.956
Flow-mediated dilation (%)	4.18 ± 2.27	5.03 ± 2.37	0.078
Reactive hyperemia index	2.00 ± 0.47	2.02 ± 0.51	0.780

Data for CD34+/CD133+/CD45^low^ cell are indicated as median value and interquartile range

*PCI* percutaneous coronary intervention, *CAG* coronary angiography, *LDL* low-density lipoprotein, *HDL* high-density lipoprotein, *eGFR* estimated glomerular filtration rate, *BNP* brain natriuretic peptide, *hs-CRP* high sensitive-C reactive protein, *ACE* angiotensin-converting enzyme, *ARB* angiotensin receptor blocker, *WBC* white blood cell

### Platelet reactivity

**Fig. 2:**
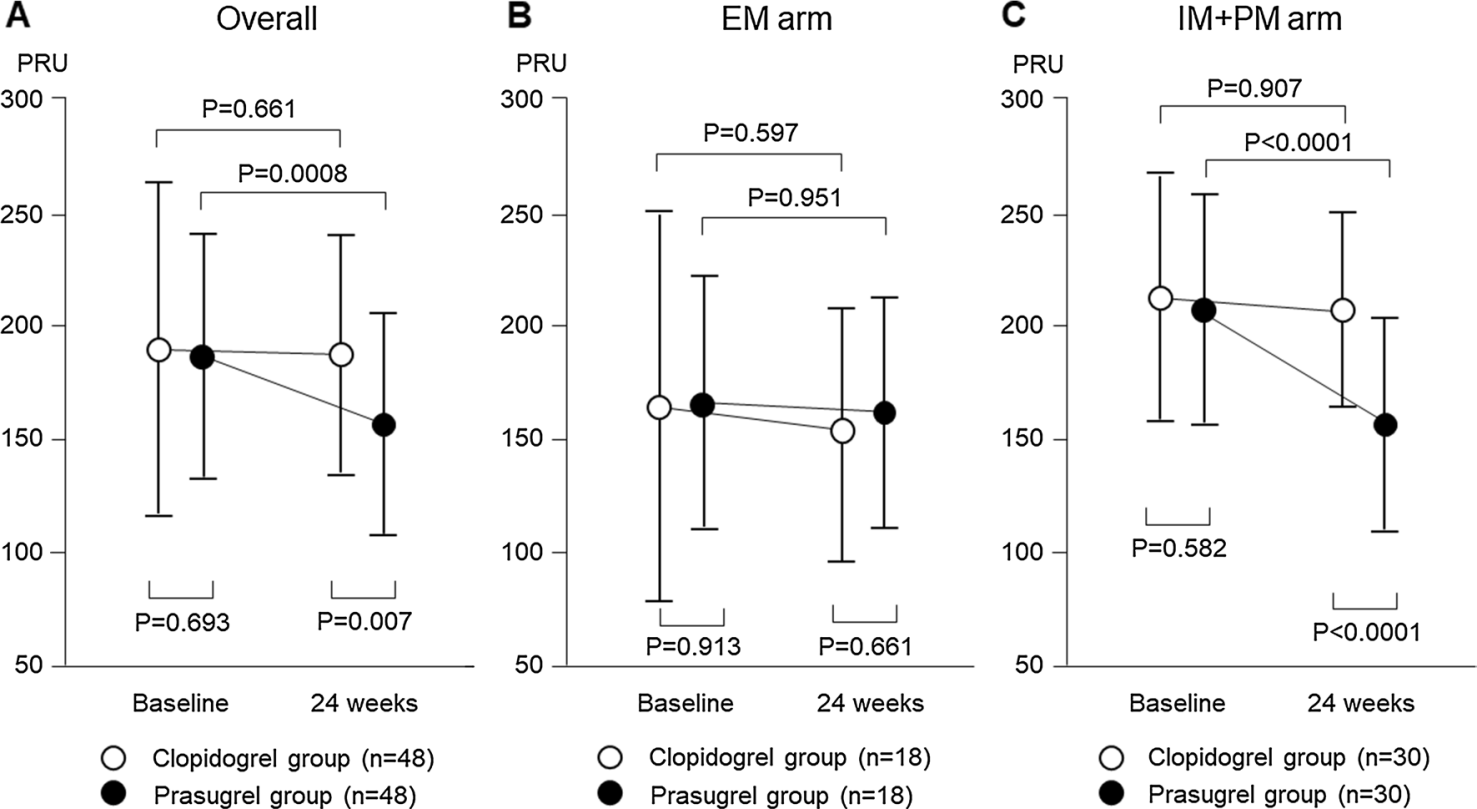
Comparison of changes in platelet reactivity between the clopidogrel and prasugrel groups. **a **Compared with baseline, the PRU value was reduced significantly at 24 weeks in the prasugrel group whereas it did not change in the clopidogrel group. The value at 24 weeks was significantly lower in the prasugrel group compared with that in the clopidogrel group. **b** The PRU value did not change significantly in the EM arm in both the clopidogrel and prasugrel groups. **c **The PRU value in the IM + PM arm was reduced significantly in the prasugrel group, but did not change in the clopidogrel group. The value at 24 weeks was significantly lower in the prasugrel group compared with that in the clopidogrel group

Compared with the baseline value, PRU was reduced significantly at 24 weeks after randomization in the prasugrel group (188 ± 58 to 157 ± 51, *P* = 0.0008), whereas it did not change significantly in the clopidogrel group (193 ± 74 to 189 ± 55, *P* = 0.661). The value at 24 weeks was significantly lower in the prasugrel group compared with that in the clopidogrel group (*P* = 0.007) (Fig. 2a). We then compared the PRU value between the clopidogrel and prasugrel groups stratified according to EM or IM + PM. This showed in the EM arm that the PRU value did not change significantly in either group (clopidogrel, 159 ± 86 to 153 ± 56; prasugrel 162 ± 59 to 161 ± 56) (Fig. 2b). In the IM + PM arm, the PRU value was reduced significantly in the prasugrel group (207 ± 51 to 154 ± 48, *P* < 0.0001), although it did not change significantly in the clopidogrel group (215 ± 55 to 212 ± 41, *P* = 0.907). The value at 24 weeks was significantly lower in the prasugrel group compared with that in the clopidogrel group (*P* < 0.0001) (Fig. 2c).

### *Circulating CD34+/CD133+/CD45*^*low*^* cells, hsCRP and vascular endothelial function*

**Fig. 3:**
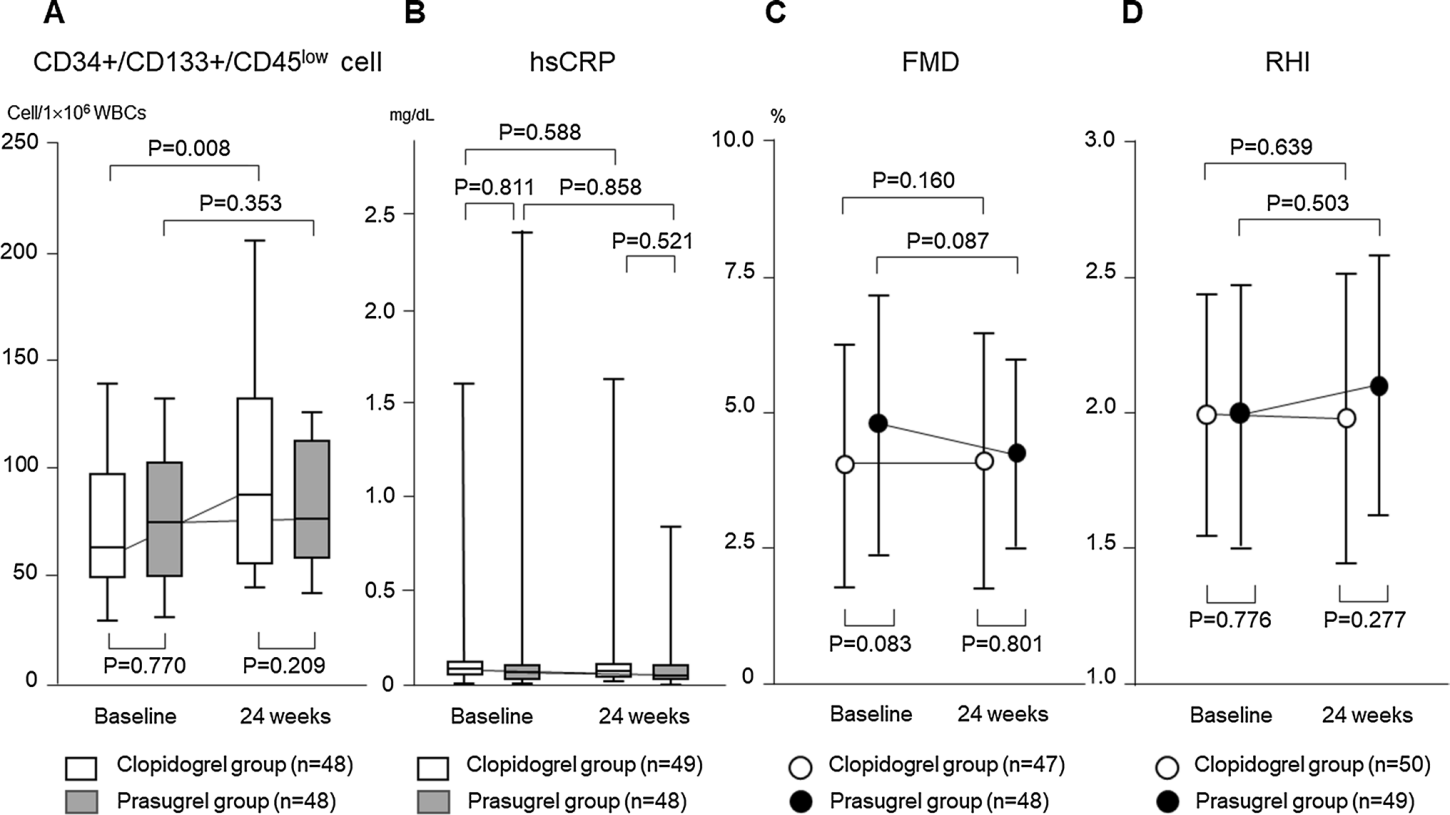
Comparison of changes in circulating CD34+/CD133+/CD45^low^ cells, serum level of high sensitivity C-reactive protein and vascular endothelial function parameters. **a** Compared with baseline, the number of circulating CD34+/CD133+/CD45^low^ cells increased significantly at 24 weeks in the clopidogrel group. However, the number of these cells did not change significantly in the prasugrel group. The number of cells at 24 weeks was compatible with the two groups. **b** The hsCRP level did not change at 24 weeks in both clopidogrel and prasugrel groups. **c** The value of flow-mediated dilation (FMD) did not change at 24 weeks in the clopidogrel and prasugrel groups. **d** The value of the reactive hyperemia index (RHI) measured by reactive hyperemia-peripheral arterial tonometry (RH-PAT) also did not change in the clopidogrel and prasugrel groups

In all patients, the number of circulating CD34+/CD133+/CD45^low^ cells increased significantly at 24 weeks after randomization in the clopidogrel group, compared with baseline [64 (50–95) to 89 (56–132) cells/1 × 10^6^ WBCs, *P* = 0.008]. However, it did not change significantly in the prasugrel group [75 (53–98) to 77 (60–110) cells/1 × 10^6^ WBCs, *P* = 0.353]. The number of cells at baseline (*P* = 0.770) as well as at 24 weeks (*P* = 0.209) was similar in the two groups (Fig. 3a). The hsCRP level did not change significantly at 24 weeks in both the clopidogrel and prasugrel groups [0.063 (0.029–0.135) to 0.058 (0.022–0.130) mg/dL, *P* = 0.588; 0.040 (0.021–0.080) to 0.040 (0.023–0.104) mg/dL, *P* = 0.858, respectively] (Fig. 3b). The FMD value did not change significantly at 24 weeks in both the clopidogrel and prasugrel groups (4.28 ± 2.27 to 4.65 ± 2.54%, *P* = 0.160; 5.15 ± 2.55 to 4.77 ± 1.82%, *P* = 0.087, respectively) (Fig. 3c). The RHI value also did not change significantly in both the clopidogrel and prasugrel groups (2.01 ± 0.47 to 1.96 ± 0.57%, *P* = 0.639; 2.02 ± 0.50 to 2.09 ± 0.50%, *P* = 0.503, respectively) (Fig. 3d).

In the limited patients belong to the IM + PM arm, the number of circulating CD34+/CD133+/CD45^low^ cells tended to increase at 24 weeks in the clopidogrel group, although there was no statistical significance, while it did not change in the prasugrel group. The hsCRP level, FMD and RHI did not change in both the clopidogrel and prasugrel groups (Table 2).

**Table 2:** Comparison of changes in circulating CD34+/CD133+/CD45^low^ cells, serum level of high sensitivity C-reactive protein and vascular endothelial function parameters in the IM + PM arm patients

	Clopidogrel			Prasugrel		
	Baseline	24 weeks	*P*	Baseline	24 weeks	*P*
CD34+/CD133+/CD45^low^ cell (cell/1 × 10^6^ WBCs)	72 (50–91)	92 (55–128)	0.081	75 (54–117)	79 (58–122)	0.745
hsCRP (mg/dL)	0.070 (0.026–0.146)	0.065 (0.022–0.143)	0.704	0.038 (0.022–0.126)	0.071 (0.017–0.129)	0.815
FMD (%)	4.00 ± 2.27	4.21 ± 1.92	0.704	5.16 ± 2.11	4.83 ± 1.73	0.52
RHI	2.00 ± 0.51	1.92 ± 0.53	0.482	1.98 ± 0.49	2.12 ± 0.51	0.302

*IM* intermediate metabolizer, *IM* poor metabolizer, *WBCs* white blood cells, *hsCRP* high sensitivity C-reactive protein, *FMD* flow-mediated dilation, *RHI* reactive hyperemia index

## Discussion

The present study demonstrated that switching from a maintenance dose of clopidogrel to that of prasugrel even during the late phase after PCI (i.e., at 24 weeks) resulted in greater inhibition of platelet reactivity, demonstrated as a reduction in the PRU value. This advantageous effect of prasugrel over clopidogrel was especially evident in the IM + PM arm but was absent in the EM arm.

Because clopidogrel is a prodrug that is biotransformed into its active moiety by cytochrome P450 enzymes, particularly CYP2C19, genetic variants of this enzyme may interfere with metabolic activation and the extent of platelet inhibition during treatment. On the other hand, prasugrel is not affected by CYP2C19 variants, because CYP3A4 and CYP2B6 are the predominant activators of prasugrel [21]. Therefore, selection of treatment in the EM, IM, or PM patients can be based on the CYP2C19 genotype, with platelet reactivity less inhibited by clopidogrel than by prasugrel in IM and PM patients. In the PRASFIT-ACS study, randomization to receive either clopidogrel or prasugrel was conducted immediately after PCI, with all the patients receiving the first loading dose and then the maintenance dose of each agent. Similar to the present study, the PRASFIT-ACS study [22] also compared the PRU value between both agents, with the data stratified into two arms (IM + PM and EM). This showed in the EM patients that prasugrel had a quicker onset of action compared with that of clopidogrel, with significantly lower PRU at 2–4 and 5–12 h after the loading dose. However, the PRU was similar in both groups from week 4 onwards. In contrast, in the IM + PM patients, the PRU was significantly lower in the prasugrel group than in the clopidogrel group throughout the study period, including 2–4 and 5–12 h after the loading dose [22]. Like the present study, Nishi et al. [23] investigated the effect of platelet reactivity inhibition by switching from maintenance clopidogrel to prasugrel. However, unlike the present study, their study was designed as a single-arm study and not a randomized group comparison study, in which clopidogrel was switched to prasugrel and at day 14 prasugrel was switched back to clopidogrel without a washout period. The results showed that prasugrel caused stronger platelet inhibition than clopidogrel. Shimamatsu et al. [24] investigated the effect on PRU in patients with long-term (median duration: 1824 days) dual anti-platelet therapy (aspirin plus clopidogrel) after PCI when switching from clopidogrel to prasugrel. Twenty-three patients with PRU ≥ 208 at enrollment were randomly assigned into either continuing to receive clopidogrel or switching to prasugrel. The PRU for IM + PM group was significantly reduced in switching from clopidogrel to prasugrel. Moreover, prasugrel showed a significant reduction in PRU value even when combined with proton pump inhibitor, esomeplazole. The results of the present study provide strong support for the findings of these earlier investigations. We propose that switching from clopidogrel to prasugrel may be a therapeutic option even during the late phase after PCI, especially in patients at higher risk of stent thrombosis.

The present study also compared the effects of clopidogrel and prasugrel treatment on the mobilization of CD34+/CD133+/CD45^low^ cells, inflammatory reaction determined by hsCRP and vascular endothelial function determined by FMD and RH-PAT. There is evidence that thienopyridine drugs have pleiotropic anti-atherosclerotic effects that include improving vascular endothelial function. A single loading dose of clopidogrel has been shown to increase FMD in patients with stable coronary artery disease [25], while a maintenance dose of prasugrel increased FMD in patients with ACS undergoing PCI to a greater extent than that observed with clopidogrel [26, 27]. Contrary to expectations, our study showed FMD and RHI values as well as hsCRP level did not change 24 weeks after randomization compared with baseline in both the clopidogrel and prasugrel groups. However, the number of circulating CD34+/CD133+/CD45^low^ cells, which we used as the EPC linage, increased significantly at 24 weeks in the clopidogrel group, but not in the prasugrel group. In the limited patients belong to the IM + PM arm, a trend of increase in the number of circulating CD34+/CD133+/CD45^low^ cells was observed at 24 weeks in the clopidogrel group, although there was no statistical significance. Our results suggest that clopidogrel might induce mobilization of EPCs, independently of platelet reactivity.

EPCs play a major role in the pathogenesis of atherosclerosis, with a decrease in the number of these cells indicating vascular endothelial injury and thereby acting as an independent predictor of morbidity and mortality of cardiovascular diseases. It is considered that the regulation of the number and function of EPCs directly influences the maintenance and development of atherosclerosis [17, 28]. Therefore, it is clinically important to increase EPC bioactivity using appropriate interventions. It has been reported that clopidogrel enhances atorvastatin-induced mobilization of EPCs as demonstrated by the presence of CD34+/CD133+/KDR + cells in patients with coronary artery disease [29]. In a rabbit model of atherosclerosis produced by a combination of a high cholesterol diet and balloon catheter-injury of the iliac artery, clopidogrel induced endothelial regeneration that involved re-endothelialization and neointimal coverage over denuded luminal surfaces of the vessel to a greater degree than aspirin and equivalent to that caused by atorvastatin [30]. EPCs contribute to re-endothelialization of injured vessels and neovascularization of ischemic tissues. In the wound healing process after PCI using stent implantation, EPCs are mobilized at the acute phase, resulting in re-endothelialization of the denuded vessel surface and neointimal coverage over the stent struts at the long-term phase. Drug-eluting stents inhibit the EPC mobilization, leading to impaired vascular healing [9, 16, 18]. We previously observed impaired vascular healing 12 months after implantation of a drug-eluting stent using optical coherence tomography and coronary angioscopy [18]. In the present study, drug-eluting stents were used in more than 66% of the patients. Therefore, in perspective of vascular healing after stent implantation, clopidogrel would be advantageous over prasugrel, since the continuation of clopidogrel compared to a switch to prasugrel promoted a subsequent increase in CD34+/CD133+/CD45^low^ cells in the present study. Since atherosclerosis is a chronically progressive disease, with reduced number and dysfunction of EPCs, clopidogrel might have a continuous potentiality to enhance the EPCs even after chronic phase after PCI.

### Potential limitation

Although the present study was designed as a prospective, randomized, group comparison study, determination of the sample size required to provide sufficient statistical power was not performed. In addition, randomization was performed using a computer-based random number table without covariate adaptation and, therefore, inter-group comparisons of baseline characteristics showed significant differences for several variables. Because our study included a small number of patients we could not set cardiovascular events as an endpoint, but alternatively assessed surrogate endpoints such as platelet reactivity and vascular endothelial function. Larger scale, event-driven trials with stratified randomization to treatment are required to verify the validity of our results.

## Clinical implication/conclusions

The duration of dual anti-platelet therapy with aspirin and thienopyridines has been widely debated since the beginning of the drug-eluting stent era. Previously long-term dual-antiplatelet therapy was required because of concerns regarding impairment of vascular healing after implantation of a drug-eluting stent and subsequent late or very late thrombosis. However, recently the duration of this therapy has become shorter with generational advances in the performance of drug-eluting stents. More recently, it has been recommended that thienopyridine, but not aspirin, should be administered as anti-platelet monotherapy following dual-anti-platelet therapy [31]. Although the present study was designed to investigate the effects of switching from clopidogrel to prasugrel for dual anti-platelet therapy during the late phase after PCI, we believe our results could be applied to long-term anti-platelet monotherapy. Our results suggest that prasugrel is potentially better than clopidogrel for preventing thrombotic events, especially in patients with IM or PM in the CYP2C19 genotype, but that clopidogrel may have some advantage over prasugrel for vascular healing after stent implantation. From our results, we envisage that proper use of any thienopyridine drug based on the CYP2C19 genotype would be clinically beneficial.
